# Artificial Intelligence Chatbot for Depression: Descriptive Study of Usage

**DOI:** 10.2196/17065

**Published:** 2020-11-13

**Authors:** Gilly Dosovitsky, Blanca S Pineda, Nicholas C Jacobson, Cyrus Chang, Milagros Escoredo, Eduardo L Bunge

**Affiliations:** 1 Palo Alto University Palo Alto, CA United States; 2 Dartmouth College Lebanon, NH United States; 3 X2AI San Francisco, CA United States

**Keywords:** chatbot, artificial intelligence, depression, mobile health, telehealth

## Abstract

**Background:**

Chatbots could be a scalable solution that provides an interactive means of engaging users in behavioral health interventions driven by artificial intelligence. Although some chatbots have shown promising early efficacy results, there is limited information about how people use these chatbots. Understanding the usage patterns of chatbots for depression represents a crucial step toward improving chatbot design and providing information about the strengths and limitations of the chatbots.

**Objective:**

This study aims to understand how users engage and are redirected through a chatbot for depression (Tess) to provide design recommendations.

**Methods:**

Interactions of 354 users with the Tess depression modules were analyzed to understand chatbot usage across and within modules. Descriptive statistics were used to analyze participant flow through each depression module, including characters per message, completion rate, and time spent per module. Slide plots were also used to analyze the flow across and within modules.

**Results:**

Users sent a total of 6220 messages, with a total of 86,298 characters, and, on average, they engaged with Tess depression modules for 46 days. There was large heterogeneity in user engagement across different modules, which appeared to be affected by the length, complexity, content, and style of questions within the modules and the routing between modules.

**Conclusions:**

Overall, participants engaged with Tess; however, there was a heterogeneous usage pattern because of varying module designs. Major implications for future chatbot design and evaluation are discussed in the paper.

## Introduction

### Background

According to the World Health Organization [1], there is a global shortage of health workers trained in mental health. Many mental health interventions do not reach those in need, with approximately 70% with no access to these services [2]. In the United States, 42.6% of adults with mental illness received mental health services in 2017 [3]. More specifically, in primary care settings, 75% of patients with depression have one or more structural or psychological barriers that interfere with access to behavioral treatments [4]. To address these challenges, Kazdin and Rabbitt [2] called for new models of delivering psychosocial interventions. Mohr et al [4] suggested that behavioral intervention technologies (BITs) offer a potential solution to overcome barriers that prevent access and expand mental health care.

BITs are the application of behavioral and psychological intervention strategies through the use of technology features that address behavioral, cognitive, and affective components that support physical, behavioral, and mental health [4]. BITs, such as internet interventions for anxiety and depression, have empirical support with outcomes similar to therapist-delivered cognitive behavioral therapy (CBT) [5]. Several BITs involve the same content as face-to-face CBT programs that allows it to reach larger numbers of people at lower costs [6].

Chatbots represent a particular type of BIT to address mental health conditions. Chatbots are computer programs that engage in text-based or voice-activated conversations [7] and that respond to users based on preprogrammed responses or artificial intelligence (AI) [8]. Ho et al [9] found that interactions with chatbots were as effective as human interactions in offering emotional, relational, and psychological benefits and that they focused on the impact of personal disclosure.

A total of 2 reviews have covered studies on mental health chatbots in mental health [10,11]. Abd-alrazaq et al [10] reported that the inconsistency of outcome measures made it difficult to compare the efficacy of chatbots. Vaidyam et al [11] reported that there is little understanding of the therapeutic effect of chatbots and a lack of consensus in the standards of reporting and evaluation. Some of the chatbots targeting mental health that have been reported in the literature are Woebot [12], Shim [13], KokoBot [14], Wysa [15], Vivibot [16], Pocket Skills [17], and Tess [18].

Woebot is an automated conversational agent designed to deliver CBT in a brief way, and it also performs mood tracking [12]. Shim focuses on positive psychology and the components of CBT [13]. KokoBot teaches cognitive reappraisal skills and facilitates peer-to-peer interactions through a postresponse platform where users post about a situation and other users respond back [14]. Wysa is an AI-based emotionally intelligent mobile chatbot aimed at enforcing mental resilience and promoting mental well-being using a text-based conversational interface [15]. Woebot, Wysa, and Shim did not provide information on how much time users spent engaging with these chatbots [12,13,15]. Vivibot [16] is a chatbot that delivers positive psychology for young individuals after cancer treatment. Finally, Pocket Skills [17] is a conversational mobile web app that supports clients with dialectical behavioral therapy.

Tess (X2AI Inc) is an automated mental health chatbot powered by AI. It engages its users with text-based conversations that deliver coping strategies based on the emotional needs of the users [18]. Research suggests that using Tess has been helpful in a variety of contexts. In a pilot study, Ackerman et al [19] found that conversations with Tess were useful in providing emotional support to a small sample (n=26) of employees in a health care system, and most participants found it helpful as Tess provided relevant support and coping tips. Fulmer et al [18] reported that using Tess helped reduce depressive and anxiety symptoms among college students (n=74) at higher rates than those in a control condition after 2 and 4 weeks of engagement with Tess. Furthermore, in a feasibility study by Stephens et al [20] with a small sample (n=23) of adolescents coping with weight management and prediabetes symptoms, the authors found conversations with Tess useful in supporting them toward their goals and high usefulness ratings.

Although findings from these studies suggest that using Tess has been effective in providing support to adults and adolescents in reducing the severity of mental health conditions, these studies do not provide information on how this chatbot works. Some chatbots include different modules (ie, preset dialogs about specific topics), and each module has different items (ie, questions or messages sent to the user). Users may follow a different path within the modules and between modules. Research that explores the potential flow of modules allows researchers to compare the treatments that are actually being delivered to users. Moreover, this flow can be helpful to determine for how long users utilize these treatments and how the AI decides to funnel users. This could prove to be insightful in both understanding what treatments are being delivered and how this flow might be further optimized. In addition, exploring item-level interactions allows researchers to gain a fine-grained understanding of how users navigate through the modules and identify when they discontinue the use of the platform. Although emerging evidence shows that chatbots may reduce symptoms and result in favorable outcomes, it is still unclear how chatbots work at the item level (within module) and module level (between modules), which represents a major limitation of chatbot research. Furthermore, there is a lack of models informing the design or implementation of BITs in general [21] and chatbots in particular. There is a need to examine how chatbots are designed and utilized at the item level and module level. Understanding the unique courses of users through Tess is a key first step in understanding how chatbots work.

### Objectives

This study attempts to understand how the chatbot Tess works, to provide a framework for future research. The first aim is to describe the overall utilization of Tess, including the total number of interactions with the depression modules, user messages, characters typed, and average time of engagement with the modules. The second aim is to understand the participant flow between the modules. The third aim is to describe the utilization of each module through the number of user messages, characters typed, average time of utilization, and completion rates. The fourth aim is to understand participant flow within modules by evaluating the number of items, duration of usage, characters used, number of messages sent, and patterns of utilization. In addition, recommendations for developers will be offered so that chatbots can be studied empirically.

## Methods

### Participants

A total of 4967 users engaged with Tess between July 27, 2017, and September 15, 2018. Of the 4967 users, 354 interacted with at least one of the 12 modules on depression, which is the sample used in this study. Users were engaged in natural conversations with Tess through Facebook Messenger, and no demographic variables were systematically collected; therefore, the demographic makeup of the sample used in this study is unknown.

### Tess

**Figure 1:**
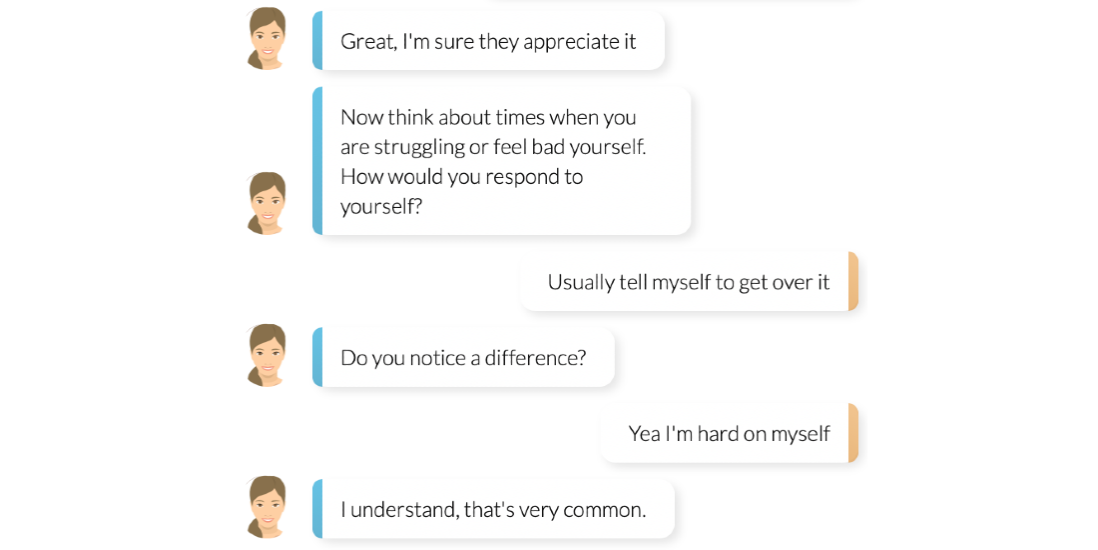
A sample transcript of a participant interacting with Tess.

Tess is a mental health chatbot designed by X2AI that is trained to react to the user’s emotional needs by analyzing the content of conversations and learning about the user that she is chatting with. Users can chat with Tess in multiple ways, such as through text message conversations or Facebook Messenger. Although Tess is designed to act like a therapist, she is not a substitute for traditional therapy. Tess’s algorithm is organized into distinct modules that focus on different types of treatment modalities and differ in content and length (Table 1). See Figure 1 for a sample transcript of a user interacting with Tess.

**Table 1:** Descriptions of the depression modules of Tess.

Module name	Treatment modality	Description	Chatbot messages per module, n
Cognitive distortion	Cognitive behavioral therapy	Provides information to identify and challenge irrational or maladaptive thought patterns	56
Self-soothing	Stress management	Education and instruction to use the 5 senses to self-soothe for stress management	33
Body scan	Stress management	Education and encouragement of body scan meditation exercises for stress reduction	33
Depression diet	Psychoeducation	Provides nutrition information such as eating tips and suggestions for increased mental well-being	27
Coping statements	Mindfulness	Guided usage of coping statements to build resilience through actively practicing nonjudgmental evaluations	25
Values	Acceptance and commitment therapy	Education and awareness of personal values to reappraise negative thoughts	21
Self-compassion	Self-compassion therapy	Encourages the use of self-compassion through guided meditation exercises and practice reminders	21
Transtheoretical	Transtheoretical model	Encourages behavior evaluation and intentional behavior change exercises	18
Self-talk	Self-compassion therapy	Encourages the use of self-compassion through positive reinforcements and self-talk	17
Thought journaling	Cognitive behavioral therapy	Education and guided instructions for utilizing thought journaling to track mood over time	17
Radical acceptance	Dialectical behavioral therapy	Education and practice using radical acceptance to build resilience against challenging situations	15
Solution focus	Solution-focused brief therapy	Encourages seeking and expanding social support for increased mental and physical health	13

### Technical Terms

#### From Tess

Messages initiated by the AI are referred to as *from Tess* in this paper. These messages are standardized, which means that they contain predetermined information and are meant to guide a user through a given intervention.

#### To Tess

Messages written by the user are referred to as *to Tess* in this paper. Data were collected on these messages, including the time when they were sent, the number of characters they contained, and the overall number of messages each user sent within a certain module and overall.

#### Routing

The flow of each module and the transition between modules is determined by the *routing* designed for Tess. Each module is made up of a set of 13 to 56 standardized messages from the AI. If within a module the user references another issue that Tess determines to be more urgent, the user will be routed to a different module.

#### Within-Module Interactions

Each module is made up of a variable number of interactions that are predesigned to fit the goals of the given module. These messages are sent by Tess to the user. The number of messages from Tess to the user for each module is shown in Table 1.

#### Between-Module Interactions

Between module is the term used to describe the transition from one module to another. Each module was considered to be an additional step that the user initiated. It is important to note that this study only analyzed data on the 12 depression modules, and it is likely that users also completed or attempted another module outside of depression.

#### Discontinued (Depression Modules)

The term *discontinued* is used in this paper to indicate when the users stopped using one of the modules for depression. It is important to note that discontinuation may be because of several reasons. Discontinuing a module may mean that the user moved to a different depression module because they or Tess found it to be more relevant at a certain time, the user moved to a nondepression module on which data were not collected in this study, or the user stopped using Tess completely. It is important to note that if a user stopped using one of the modules, they did not necessarily stop using Tess overall. With these data, the explanation for user discontinuation is not known.

### Procedures

The Institutional Review Board determined that this study is not human subject research. All the users in this study used Tess through Facebook Messenger. Users interacted with Tess for free and were not compensated. Users could find Tess through social media advertisements. Once users got to Tess, they provided written consent to participate in the study.

When users report depression, Tess selects 1 of the 12 modules for treating depression based on the conversations that she has had with other users, previous (if any) conversations with the user, and other information that is input into an algorithm.

### Analyses

Descriptive statistics were used to analyze the overall chatbot usage and module usage. Data collected included total messages sent by the user to Tess, total messages sent by Tess to the user, total characters typed by the user, and duration of usage. The overall chatbot usage of the depression modules was analyzed using slide plots [22] that were created from the messages sent to and from Tess. These slide plots show the sequence between the depression modules, such as where the users started, where the users were directed to, and where the users discontinued. The slide plots show the aggregated trajectories of individuals. The thickness of a segment is proportional to the frequency of transition from one state to another.

Descriptive statistics were used to analyze how participants utilized each depression module. Means, SDs, and ranks for characters per message to Tess; completion composites; and time spent on each module are reported. Characters per message were used over characters or messages separately to account for the variance in module length. Completion composite scores were calculated for each module. The composite was calculated by multiplying the proportion of users who completed each module by the number of module interactions. The completion composite was used in favor of simply using the proportion completed to account for differences in length between modules. For the time spent per module, when there was a period of inactivity of more than 2 SDs above the mean, those periods were excluded from the calculation. This was done because conversations with a chatbot tend to be asynchronous; therefore, long breaks between user messages are expected. Slide plots were also used to analyze the flow of each depression module individually.

## Results

### Overall Utilization of Tess

Descriptive statistics were used to analyze the data from the messages sent by the participants to Tess. The 354 participants included in this study had at least one interaction with one of the modules (mean 2.18, SD 1.56; range 1-10 modules) and sent a total of 6220 user messages (mean 17.57, SD 19; range 1-73 messages) with a total of 86,298 characters (mean 243.78, SD 299.29; range 2-1644 characters). The average duration for which the participants engaged with the depression modules was 46 days (range 1-314 days) during the 14-month period of data collection, and the duration for which they engaged in conversations with the depression modules was 24 min and 49 seconds.

### Users Flow Between Modules

**Figure 2:**
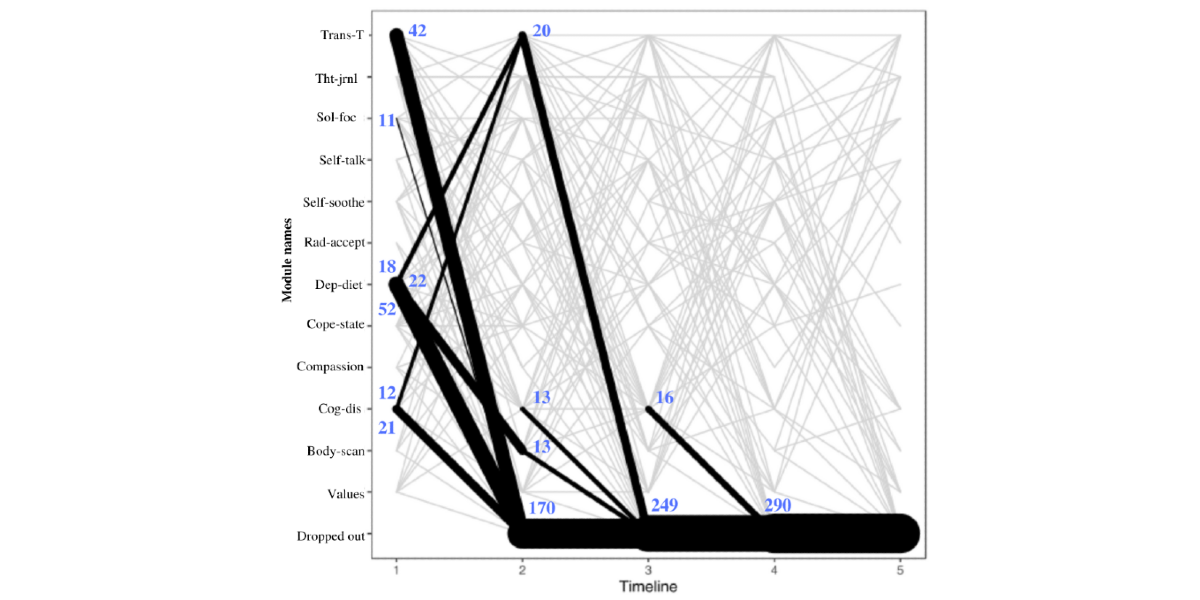
Modules initiated by Tess. The figure presents the first 5 module steps that participants interacted with that were initiated by Tess. The timeline reflects the module number to which the participant was exposed (ie, timeline 1 is the module that people started with, and timeline 2 is the second module that participants began). The line thickness describes the transitions from one module to the next and not the number of users in a given module. Body-scan: body scan; Cog-dis: cognitive distortion; Compassion: self-compassion; Cope-state: coping statements; Dep-diet: depression diet; Rad-accept: radical acceptance; Self-soothe: self-soothing; Sol-foc: solution focus; Tht-jrnl: thought journaling; Trans-T: transtheoretical.

**Figure 3:**
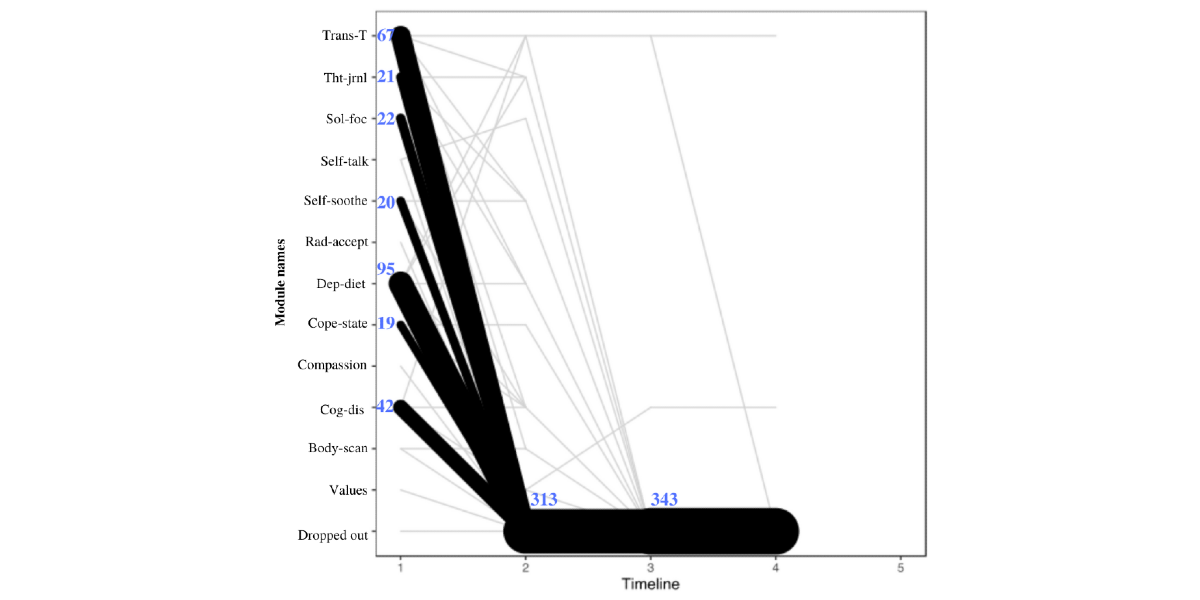
Modules initiated by user. The figure presents the first 5 module steps that participants interacted with that were initiated by the participant. The timeline reflects the module number to which the participant was exposed (ie, timeline 1 is the module that people started with, and timeline 2 is the second module that participants began). The line thickness describes the transitions from one module to the next and not the number of users in a given module. Body-scan: body scan; Cog-dis: cognitive distortion; Compassion: self-compassion; Cope-state: coping statements; Dep-diet: depression diet; Rad-accept: radical acceptance; Self-soothe: self-soothing; Sol-foc: solution focus; Tht-jrnl: thought journaling; Trans-T: transtheoretical.

To understand the participant flow through the depression modules of Tess, the sequence of interactions with the modules was analyzed using 2 criteria. The first criterion utilized was the messages from Tess (Figure 2), and the second criterion utilized was the messages to Tess (Figure 3). When using messages from Tess, most users started with the depression diet module (112/354, 31.6% users), and then they were directed to the body scan module (22/112, 19.6% users) and the transtheoretical module (18/112, 16.1% users). When using messages to Tess, most users started with the depression diet module (103 users) and then discontinued. Both Figures 2 and 3 are only representative of the sequence between depression modules. This does not account for nondepression modules completed.

From the modules initiated by Tess (Figure 2), the first pattern observed was that in step 1 most users (112/354 users, 31.6%) were routed to the depression diet module, from which 46.4% (52/112) users discontinued, 19.6% (22/112) user were routed to body scan module, and 16.1% (18/112) users were routed to the transtheoretical module in step 2. Among those who started cognitive distortion module at step 1 (49/354, 13.8% users), 24% (12/49) users went to transtheoretical module and 42% (21/49) users discontinued at step 2. Among those who started transtheoretical module at step 1 (79/354, 22.3% users), 53% (42/79 users) discontinued at step 2. Parallel lines in Figure 2 indicate that the user repeated the same module in sequence. From the modules initiated by users, 88.4% (313/354 users) discontinued by module step 2 (Figure 3).

### Utilization of Each Module

The total number of characters typed per message across the 12 modules was 11,948.66, with an average of 995.72. Across the 12 modules, the average completion rate was 40%, and the total time spent was 146 hours, 23 min, and 36 seconds, with an average of 12 hours, 11 min, and 58 seconds per module.

The module with most characters typed per message was the self-compassion module (n=10; mean 39.18; SD 15.59), followed by the transtheoretical module (n=139; mean 24.27). Although the self-compassion module had the most characters typed per message, it had the lowest number of users who had at least one interaction with this module. The module with the least number of characters typed per message was the coping statements module (n=45; mean 8.46; Table 2).

**Table 2:** Characters per message (N=354).

Rank^a^	Module	Users with at least one interaction with the module, n (%)	Total characters per message, n	Characters per message, mean (SD)
1	Self-compassion	10 (2.8)	391.82	39.18 (15.59)
2	Transtheoretical	139 (39.3)	3373.8	24.27 (16.41)
3	Values	38 (10.7)	792.19	20.85 (14.83)
4	Thought journaling	70 (20.2)	1335.74	19.08 (11.49)
5	Self-talk	39 (11.0)	742.53	19.04 (16.87)
6	Depression diet	145 (41.0)	1924	13.27 (9.79)
7	Solution focus	49 (13.8)	607.14	12.39 (18.52)
8	Cognitive distortion	116 (32.8)	1248	10.76 (8.45)
9	Self-soothing	52 (14.7)	520	10 (12.86)
10	Radical acceptance	22 (6.2)	213.6	9.71 (11.98)
11	Body scan	46 (12.9)	419.21	9.11 (5.1)
12	Coping statements	45 (12.7)	380.63	8.46 (23.22)

^a^Rank is based on the average number of characters per message for each module, with higher characters per message associated with a higher rank.

The module with the highest completion composite was the cognitive distortion module (19.79), with 35.3% (41/116) of users that interacted with the module, completed it. The module with the lowest completion composite was the transtheoretical module (5.31), with 29.5% (41/139) of users that interacted with the module, completed it (Table 3).

**Table 3:** Completion composite of users that interacted with the modules.

Rank^a^	Module	Users who had at least one interaction with the module (N=354), n (%)	Users who completed the module		Proportion completed^b^ (%)	Completion composite^c^
			n (%)	N		
1	Cognitive distortion	116 (32.7)	41 (35.3)	116	35.3	19.793
2	Body scan	46 (12.9)	24 (52.2)	46	52.2	17.217
3	Self-compassion	10 (2.8)	6 (60.0)	10	60.0	12.600
4	Radical acceptance	22 (6.2)	18 (81.8)	22	81.8	12.273
5	Self-soothing	52 (14.7)	17 (32.7)	52	32.7	10.788
6	Thought journaling	70 (19.7)	41 (58.6)	70	58.6	9.957
7	Self-talk	39 (11.01)	21 (53.8)	39	53.6	9.154
8	Coping statements	45 (12.7)	16 (35.5)	45	35.6	8.889
9	Depression diet	145 (40.9)	42 (28.9)	145	29.0	7.821
10	Solution focus	49 (13.8)	29 (59.2)	49	59.2	7.694
11	Values	39 (11.0)	13 (33.3)	39	33.3	7.000
12	Transtheoretical	139 (39.3)	41 (29.5)	139	29.5	5.309

^a^Rank is based on the completion composite, with higher completion composite scores associated with a higher rank.

^b^The proportion completed represents the ratio of users who completed each module to the users who had at least one interaction with the module.

^c^The completion composite was calculated by multiplying the proportion completed by the number of interactions in each module to account for the differences in module length.

The module with the most time spent was the values module, with users spending an average of 58 min and 29 seconds. The second module with the most time spent was the cognitive distortion module, with users spending an average of 26 min and 22 seconds. Users spent the least amount of time on the radical acceptance module, spending an average of 1 min and 52 seconds (Table 4).

**Table 4:** Time (N=354).

Rank	Module	Users who had at least one interaction with the module, n (%)	Total time^a^	Time, mean (SD)	Time, median
1	Values	23 (6.5)	22:25:08	0:58:29 (2:41:31)	0:06:11
2	Cognitive distortion	109 (30.8)	48:20:42	0:26:22 (0:57:57)	0:09:10
3	Body scan	46 (12.9)	10:44:18	0:14:00 (0:58:37)	0:02:54
4	Thought journaling	66 (18.6)	12:57:38	0:11:47 (0:20:59)	0:05:04
5	Self-soothing	48 (13.5)	9:05:25	0:11:22 (0:24:32)	0:04:16
6	Coping statements	42 (11.9)	7:42:31	0:11:01 (0:18:10)	0:04:37
7	Depression diet	132 (37.3)	16:24:04	0:07:27 (0:15:46)	0:03:06
8	Transtheoretical	121 (34.2)	13:55:02	0:06:54 (0:09:18)	0:04:15
9	Self-compassion	8 (2.3)	0:31:51	0:03:51 (0:04:35)	0:02:35
10	Solution focus	48 (13.6)	2:12:31	0:02:46 (0:06:50)	0:01:20
11	Self-talk	31 (8.8)	1:23:30	0:02:42 (0:02:30)	0:01:39
12	Radical acceptance	22 (6.2)	0:40:56	0:01:52 (0:01:14)	0:01:34

^a^Total time was calculated as the duration, in hours, between the first message sent by the user and the last message sent by the user in each module. When there was a period of inactivity of more than 2 SDs above the mean, those time periods were excluded from the calculation. Time is presented in the format hh:mm:ss.

The modules with larger sample sizes were the depression diet (n=145), transtheoretical (n=139), and cognitive distortion modules (n=116; Table 2). The depression diet module was ranked sixth for the number of characters typed per message, ranked ninth for completion, and ranked seventh for time. The transtheoretical module was ranked second for the number of characters typed per message, ranked 12th for completion, and ranked eighth for time. The cognitive distortion module was ranked eight for the number of characters typed per message, ranked first for completion, and ranked second for time.

### User Flow Within Modules

Each of the 12 modules was unique in terms of the number of questions it had, duration of usage by the user, characters used, messages sent, and completion rate. The content of each module also differed in terms of the type of questions and messages used by Tess as well as the utilization of links that direct users to leave the platform.

**Figure 4:**
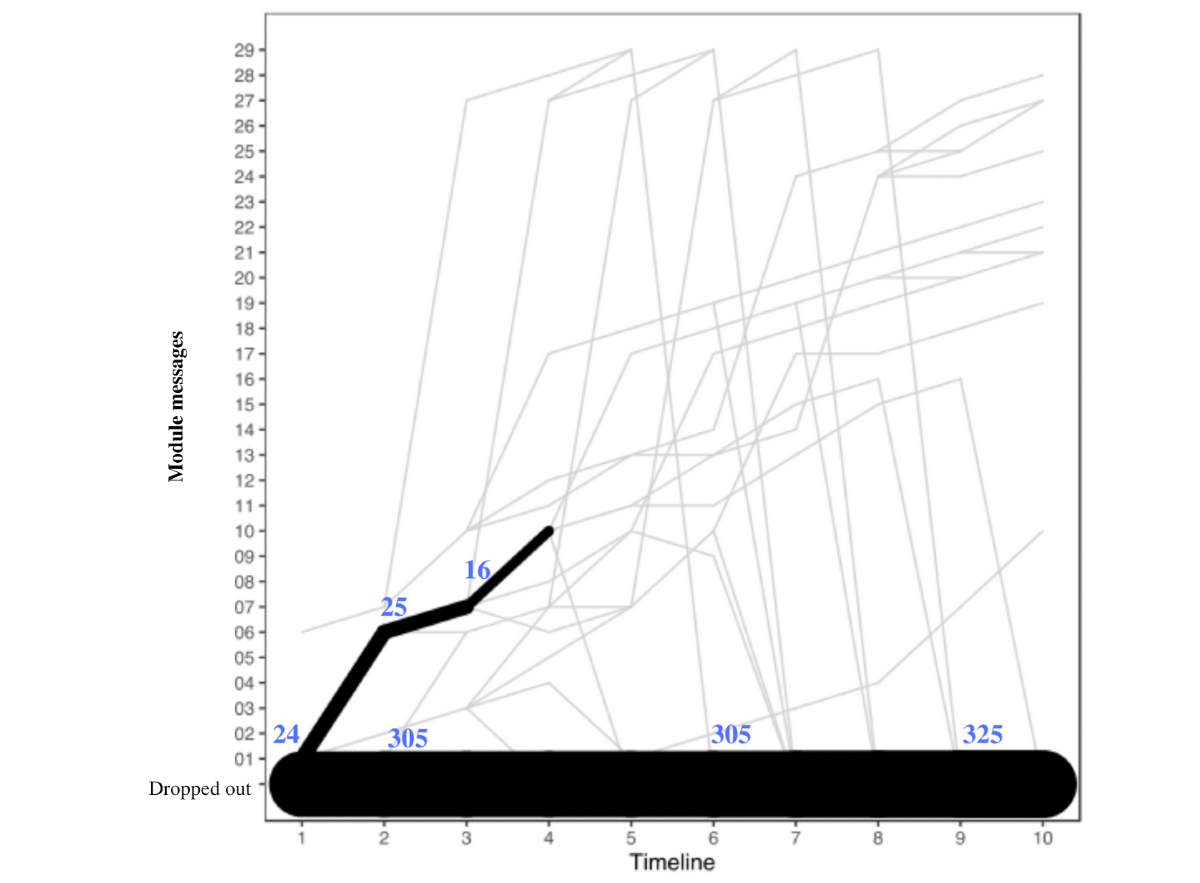
User flow through the body scan module. The timeline reflects the module number to which the participant was exposed. Frequencies are based on messages sent by the participant in association with each module question sent by Tess. The line thickness describes the transitions from one module to the next and not the number of users in a given module.

**Figure 5:**
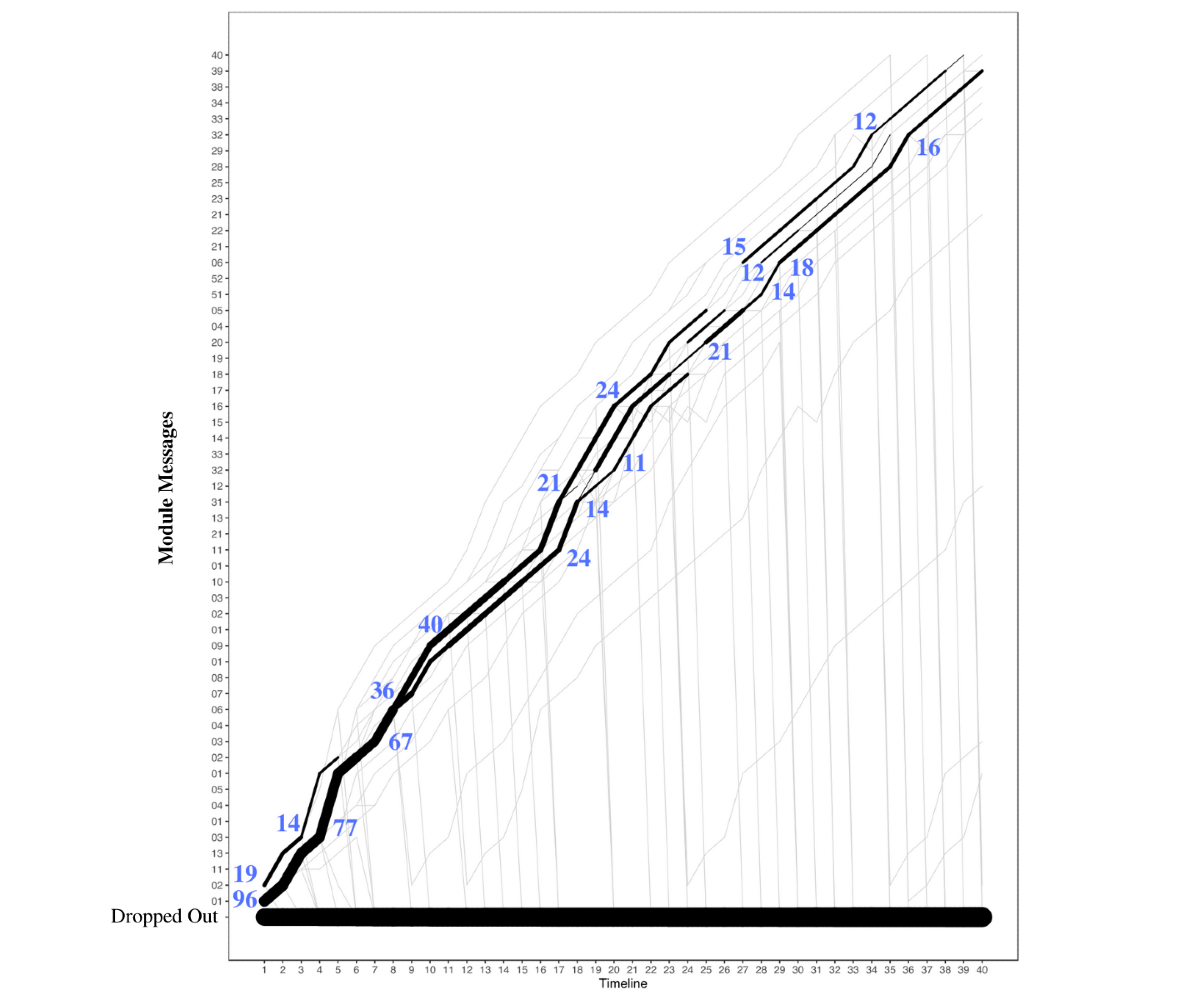
User flow through the cognitive distortion module. The timeline reflects the module number to which the participant was exposed. Frequencies are based on messages sent by the participant in association with each module question sent by Tess. The line thickness describes the transitions from one module to the next and not the number of users in a given module.

Overall, 2 of the 12 modules were selected to highlight the possible differences that can be seen when evaluating modules that were created in different ways. The body scan and cognitive distortion modules differed most noticeably in terms of duration. The body scan module had 33 messages from Tess (Figure 4), whereas the cognitive distortion module had 56 messages (Figure 5). In addition, the body scan module included links that directed users to leave the platform at several points.

The body scan module shows a heterogeneous pattern of usage that shows that most users were branched to different items and that there were few consistent transition points, whereas the cognitive distortion module did not have much branching, as most users were routed through the module in a consistent and linear way.

## Discussion

### Principal Findings

Although previous studies on chatbots show promising results, little is known about how chatbots work. Understanding how chatbots work, especially chatbots that utilize modules as Tess, is essential for researchers to compare the treatments that are actually being delivered and provide guidance on how chatbots could be designed. Studying how users engage with the modules and the aspects of modules that are associated with completion or engagement can help future chatbot developers. This study is a first attempt to understand how a specific chatbot (Tess) works, including the organization by modules, module length, and other characteristics, and to provide a framework for future chatbot research.

The first aim of this study is to understand the overall utilization of the depression modules from Tess. This was done by analyzing the number of user messages, characters per message, the average time of utilization, and participant flow through Tess. The 354 users included in this study sent a total of 6220 messages and typed a total of 86,298 characters across an average of 46 days, which illustrates that the users engaged with Tess. However, when the time spent was analyzed, the participants engaged with Tess for an average of 25 min. A cautious interpretation of this number may suggest that 25 min is not sufficient to provide strategies that can help users cope with depression. A more enthusiastic interpretation suggests that as chatbots are highly accessible and scalable, 25 min of an asynchronous conversation delivered right when users need it can help boost their mood, and if this is delivered to large populations, this could be a major contribution to the mental health resources.

With regard to the second aim, when analyzing the participant flow between the depression modules, the sequence of interactions with the modules was heterogeneous, and users were more likely to engage with modules when they were initiated by Tess rather than by the user (Figures 2 and 3). In addition, most users discontinued the depression modules after completing 1 to 2 modules. Although there might be several reasons for why the participants discontinued the module (eg, not being depressed any more or not being interested in the module; refer to the *Limitations* section), chatbots researchers should keep in mind that the attrition problems of most digital interventions are still present with chatbots [23]. Thus, when developing a chatbot, it may be better to focus first on developing a few good modules, rather than many modules that may not be used or comparable.

The third aim of this study is to compare the utilization patterns across the depression modules based on the messages sent, characters typed, completion rate, and average time of utilization. The results showed that the overall utilization was heterogeneous across modules. More specifically, the differences between characters typed per message and the average time of utilization across modules may be because of the differences in how the modules were designed. Most probably, user engagement changes depending on what type of messages the AI sends to the user. For example, a module that uses more open-ended questions may trigger more characters to be typed than a module that uses close-ended questions that elicit yes or no answers from the users.

The overall completion rate of 40% may be considered as a good engagement level for digital interventions [24-27], especially considering that AI may redirect users to more relevant modules as they chat. For users to whom the module was not relevant, being directed away from the module actually indicates the effectiveness of the AI. This should be considered when evaluating the completion rate as a measure of AI usability. In addition, the relation between completion rate and composite score (which accounts for the number of interactions) may yield useful information. For example, the radical acceptance module had the highest completion rate but was ranked third based on the completion composite because this module had 15 interactions, as it was one of the shorter modules. The cognitive distortion module was the longest module and had one of the lowest completion rates. When evaluating completion, one aspect to be considered is the balance between the complexity of the conversation and the user experience. It may be difficult to present complex concepts in a succinct manner. At the same time, if users do not finish the conversation, their experience may be less positive. Therefore, assessing completion rates using a holistic approach, rather than as an isolated variable, may be more appropriate. For example, completion could be assessed by integrating completion rates, composite scores, number of characters typed, time spent in conversation, and the duration required to communicate specific concepts (a problem-solving explanation may be shorter than an explanation of cognitive distortions).

With regard to the time of utilization, the engagement with each module was variable, ranging from 31 min to 48 hours. As interactions with chatbots are asynchronous, users may engage with each module over the course of a day, week, or longer, and determining what period of inactivity indicates that the user is no longer engaged in a module leads to arbitrary decisions. Therefore, judging the time spent with the module may be limited. Although the time spent in a conversation should be considered as a measure of the usability of a chatbot, it is unclear as to what would be the best way to measure it. One possibility is that a composite score based on the time spent, number of sessions initiated, number of sessions per day, and number of days (from the first session to the last session) could yield more meaningful information.

Overall, the wide heterogeneity in both the design and usage patterns suggested that there were no typical patterns of user engagement (as measured by the characters typed, messages sent, completion, and time) across modules. This is because each module was constructed differently (eg, differing in length, type of questions). To assess if a module achieves a good level of usability, the design of modules should be comparable.

The fourth aim of this paper is to describe participant flow within each module. In this case, 2 of the 12 modules were selected to highlight the possible differences that could be seen when evaluating modules that were created in different ways. The body scan module included links that directed users to resources outside Tess at several points. This amount of branching may be one of the explanations for why there were much fewer consistent transition points with the body scan module compared with the cognitive distortion module. The cognitive distortion module did not have much branching and had no links to external sources; consequently, most users were routed through the module in a consistent way.

### Limitations

There were several limitations to this study. First, no demographic information was collected; therefore, the demographic makeup of the sample analyzed was unknown. The usage patterns were heterogeneous, but the source of the heterogeneity is unknown (ie, because of differences among modules or differences among sample characteristics). Second, the data set did not contain information about whether a user discontinued the usage of Tess entirely or if they were redirected to a nondepression module. There are several reasons for a user to discontinue a module. They may not complete a module simply because they do not like it and decide to stop or not respond to Tess. They can also be redirected to another module partway through if the user mentions a more urgent topic. In addition, not all users who begin a module will necessarily benefit from it; therefore, if Tess realizes that a user’s responses indicate that they do not need a module, the user may be directed to a different module without completing the current one. Moreover, system errors were found for module instances from 2 users. Third, given the asynchronous nature of chatbots, it was not possible to know the precise time at which a user was actively engaged with the chatbot (no data were collected about when the user viewed the messages; only data for when they sent them were collected). Fourth, the notion of modules does not apply to all chatbots; therefore, these recommendations would not generalize to every chatbot.

### Guidelines for Developing and Assessing a Chatbot

Developers of future mental health chatbots may benefit from some insights gathered from this study. Given the scant literature on how chatbots work and are utilized, it is important to highlight that developing an engaging chatbot may be the first step to assess its efficacy. For such purposes, a table containing 6 preliminary recommendations on how to develop engaging chatbots is presented in Table 5. Due to the limitations of this study, this is not an exhaustive list but can provide guidance to those attempting to develop chatbots for mental health. Together with the recommendations, the rationale is also presented in Table 5. The list includes 5 recommendations specifically for the development of chatbots and 1 recommendation oriented to assessing the usability and engagement of the chatbot.

**Table 5:** Guidelines for developing engaging chatbots.

Tip	Definition	Rationale
Focus on the first module	Emphasis should be placed on the development of the first module to increase the chance of participants continuing to engage with the chatbot.	Most participants use only 1 module (or 2). It is important to provide the best intervention possible from the beginning.
Start small	Developers should focus on creating a few engaging and effective modules at the beginning rather than developing a large variety of untested modules.	On the basis of participant’s flow through Tess, most individuals tend to discontinue after the second module.
Develop comparable modules	To compare module utilization, modules should be built with similar characteristics (eg, length, number and type of questions, and the inclusion of links).	Utilization of modules that differ in characteristics may not allow for meaningful comparisons.
Balance length and complexity	Modules with fewer interactions may have better completion, but more complex topics may need longer modules. Developers should strive to find a module length that enhances intervention fidelity without compromising engagement.	Modules that had more interactions from the artificial intelligence had lower completion rates.
Be aware of personalization and standardization	Standardization provides streamlined data and requires less work, whereas personalization has more complexity and consequently requires more effort both in development and analysis. However, personalization may provide richer interactions.	Without understanding the efficacy of the overall modules, it is difficult to assess whether branching promoted engagement.
Holistic assessment	There is no single variable that can provide an accurate measure of utilization, rather a combination of such variables can provide a broad idea of general utilization and specific information regarding specific aspects of the intervention.	Number of messages, characters used, time spent, and completion should be considered alongside helpfulness and satisfaction in the assessment of chatbots.

As an overall recommendation at the initial stages of development, developers should focus on small steps, test them with small iterative studies until a satisfactory level of engagement is achieved, and then move toward expanding the content (or modules) of the chatbot. As with most digital interventions, the attrition rates are significantly high; therefore, developing an extensive set of modules that users do not end up engaging with is not a good use of the resources. In addition, focusing on the initial modules and developing modules that are consistent may help developers and researchers to understand and compare how users respond to the modules.

### Future Directions

The goal of research on chatbots should help researchers answer the question on the usage of specific chatbots (or modules), the people to whom they would be helpful, the circumstances under which they can be used, and how they can be used, as in psychotherapy research [28]. So far, the research conducted on chatbots does not allow for strong conclusions about the usability and efficacy of mental health chatbots or their outcomes. There are several variables that should be considered in future research on chatbots.

Usability and efficacy should be evaluated together because the process of how individuals use the chatbot (similar to process research in psychotherapy) is as important as their outcomes (similar to efficacy studies on psychotherapy), and both combined will allow for a faster pace of improvement. With regard to the process of how chatbots work, a Markov chain analysis can be utilized to predict the probability of a user completing a certain module based on their previous responses. In addition, the efficacy of the modules should be evaluated subjectively (eg, using net promoter scores, “would you recommend this to a friend”) and objectively (ie, comparison of scores on PHQ-9 (Patient Health Questionnaire-9) before and after engagement with the chatbot). Finally, research should also examine the factors that predict chatbot satisfaction and efficacy.

### Conclusions

Research on chatbots is in the initial stages, and although findings show that chatbots can be effective, more information is needed on how they work. This study showed that although many individuals used the chatbot, there was large heterogeneity in user engagement across different modules, which appeared to be affected by the length, complexity, content, and style of questions within the modules and routing between modules. At the initial stages of mental health chatbot research, developers should aim to reach acceptable levels of usability and then focus on efficacy. To increase usability and engagement, the focus should be on developing short, simple, and consistent modules and testing them with small iterative studies. Then, developers can move toward expanding the content (or modules) of the chatbot. As with most digital interventions, the attrition rates are significantly high; therefore, developing an extensive set of modules that users do not end up engaging with is not a good use of resources. Research on frameworks for developing engaging and effective chatbots offers the opportunity to create and test scalable interventions. Data from large studies on chatbots could lead to effective personalized interventions that could eventually answer the question of which intervention works for which individual.

## Abbreviations

AI: artificial intelligence
BITs: behavioral intervention technologies
CBT: cognitive behavioral therapy
